# Plant Growth Absorption Spectrum Mimicking Light Sources

**DOI:** 10.3390/ma8085240

**Published:** 2015-08-13

**Authors:** Jwo-Huei Jou, Ching-Chiao Lin, Tsung-Han Li, Chieh-Ju Li, Shiang-Hau Peng, Fu-Chin Yang, K. R. Justin Thomas, Dhirendra Kumar, Yun Chi, Ban-Dar Hsu

**Affiliations:** 1 Department of Materials Science and Engineering, National Tsing Hua University, Hsinchu 30013, Taiwan; E-Mails: chingchiao.lin@gmail.com (C.-C.L.); kratos0083@gmail.com (T.-H.L.); panther780322@gmail.com (C.-J.L.); tail523.oled@gmail.com (S.-H.P.); richkissfather@gmail.com (F.-C.Y.); 2 Organic Materials Laboratory, Department of Chemistry, Indian Institute of Technology Roorkee, Roorkee-247 667, India; E-Mails: krjt8fcy@iitr.ac.in (K.R.J.T.); dhiruorganickd37@gmail.com (D.K.); 3 Department of Chemistry, National Tsing Hua University, Hsinchu 30013, Taiwan; E-Mail: ychi@mx.nthu.edu.tw; 4 Department of Life Science, National Tsing Hua University, Hsinchu 30013, Taiwan; E-Mail: bdhsu@life.nthu.edu.tw

**Keywords:** organic light emitting diode, photosynthetic action spectrum, plant-growth light

## Abstract

Plant factories have attracted increasing attention because they can produce fresh fruits and vegetables free from pesticides in all weather. However, the emission spectra from current light sources significantly mismatch the spectra absorbed by plants. We demonstrate a concept of using multiple broad-band as well as narrow-band solid-state lighting technologies to design plant-growth light sources. Take an organic light-emitting diode (OLED), for example; the resulting light source shows an 84% resemblance with the photosynthetic action spectrum as a twin-peak blue dye and a diffused mono-peak red dye are employed. This OLED can also show a greater than 90% resemblance as an additional deeper red emitter is added. For a typical LED, the resemblance can be improved to 91% if two additional blue and red LEDs are incorporated. The approach may facilitate either an ideal use of the energy applied for plant growth and/or the design of better light sources for growing different plants.

## 1. Introduction

Plant factories have attracted increasing attention for being capable of producing fresh fruits and vegetables free from pests and pesticides in all weather in nearly all locations, including ocean vessels and space stations [1,2,3,4]. Although the use of artificial light such as torch light to trigger an early blossom had been reported in ancient China nearly a thousand years ago [5], intensive applications of plant factories have not significantly become popular until 1980s, mainly due to high energy consumption [6,7,8]. In addition to high energy consumption, much of the energy has been wasted on generating excessive emission spectrums, mismatching what plant growth truly needs.

Beside the light-absorbing and photosynthesis-active chlorophyll-a, plant growth needs chlorophyll-b to assist light absorption for chlorophyll-a and carotenoids to also assist light absorption and further release any excessive photonic energy that might damage chlorophyll-a and chlorophyll-b. The ideal light for plant growth should hence have, at least, an emissive spectrum covering those three pigments. To grow plants in an energy-efficient manner in practice, the emissive spectrum should closely match the three pigments containing the photosynthetic action spectrum (PAS) observed from a chloroplast [9]. Developing a lamp covering the PAS will enhance the growth of the plants according to the study by Singhal *et al*. [9]. The photosynthetic action spectrum needed may vary with different plants and/or with variations in growing roots, stems, leaves, and fruits in different seasons and/or at different times diurnally. As a result, the ideal lighting device should also possess a high degree of design freedom in spectrum-tailoring so that the resultant spectrum can better match chlorophyll-a, chlorophyll-b, and carotenoids, individually or collectively, to realize economical plant growth or to further understand the effects of light on the growth of various plants for varying purposes.

Nevertheless, few emission spectra from current light sources, including high pressure sodium (HPS) lamps, incandescent bulbs, fluorescent tubes, and light-emitting diodes (LED), closely match the photosynthetic action spectrum. For example, the resemblance is only 38% between the emission spectrum of a high pressure sodium lamp with the PAS, while it is 50%, 60%, and 58% for an incandescent bulb, a fluorescent tube, and a plant factory light-emitting diode (Table S1), respectively. To improve on this, several research groups reported on a combination of fluorescent tubes with incandescent bulbs or red LEDs with blue fluorescent tubes being employed [10,11,12,13,14]. According to these data, the resemblance is still quite low due to low flexibility in tailoring their emission spectra.

In contrast, organic light-emitting diodes (OLEDs) possess very high spectrum-tailoring flexibility because there is a wide variety of emitters ranging from red to violet or even from infrared to ultra-violet, and their chromaticity can further be tuned via molecular designs [15,16,17,18,19], microcavity technologies [20,21,22,23,24], and/or device engineering [25,26,27,28]. Furthermore, the inherently diffused emission of organic emitters enables OLEDs to generate a desirable multiple broad-band spectrum closely matching the intrinsically diffused blue and red bands in the PAS. OLEDs are also plane light sources, just like that of the sky. Their emitting areas can be as large as 30 cm by 30 cm, and they emit steady and soft lights for growing plants [29]. The emission from LEDs is typically very sharp. For example, the FWHM (full width at half maximum) of a typical blue LED is 25 nm while it is 100 nm for a blue OLED counterpart. This explains why even the plant factory LED lamps showed just a fair PAS resemblance. However, introducing multiple broad-band emissions into LEDs can greatly enhance their resemblance while retaining the advantages of high efficacy and high reliability.

In this study, we demonstrate a design concept by using multiple broad-band as well as narrow-band solid-state lighting technologies to design plant growth light sources. Among these, OLEDs can closely mimic almost any natural light with any desirable color [30]. The resultant OLED device shows to be an ideal light source for plant growth, as confirmed via the theoretical calculations. It is because organic electro-luminescent materials can emit any color throughout the entire visible region, and their spectra are broad and diffused, where the electro-luminescence is defined as an optical and electrical phenomenon in which an organic material emits light in response to the passage of an electric current or to a strong electric field. As a result, plant growth light sources with different absorption colors can be synthesized with the employment of a low number of OLED emitters.

## 2. Experimental Section

**Figure 1:**
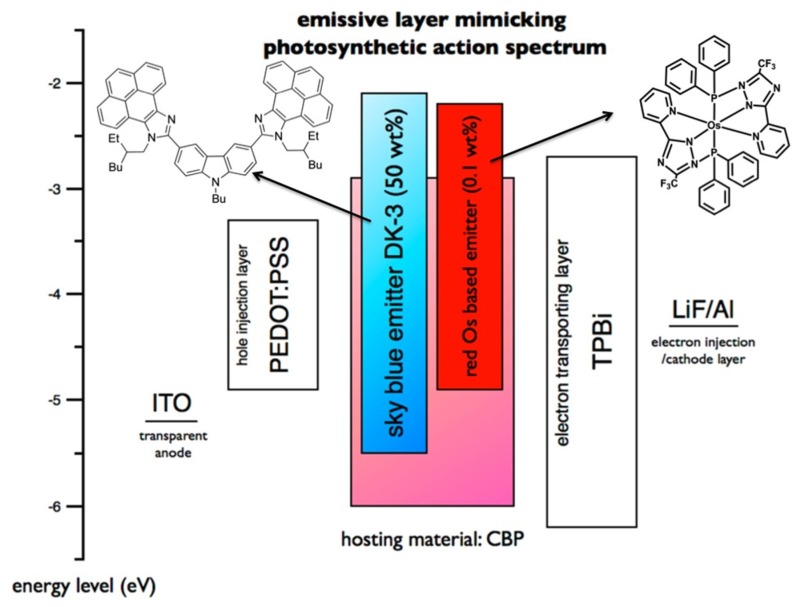
Schematic illustration of the photosynthetic action spectrum-mimicking OLED device that is composed of a single solution-processable emissive layer with a sky-blue emitter and a red emitter dispersed in a host and their molecular structures. Notably, emission tuning from light- to deep-blue can be done by simply varying the doping concentration of the sky-blue emitter.

Figure 1 shows the device structure and its corresponding energy level diagrams of the OLED device. The device structure was composed of a 125 nm indium tin oxide anode layer (ITO), a 35 nm poly(3,4-ethylene-dioxythiophene)-poly-(styrenesulfonate) (PEDOT:PSS) hole injection layer, a 45 nm photosynthetic action spectrum mimicking emissive layer, a 32 nm 1,3,5-tris(*N*-phenylbenzimidazol-2-yl)benzene (TPBi) electron transporting layer, a 0.7 nm lithium fluoride (LiF) electron injection layer, and a 150 nm aluminum cathode layer. The emissive layer consisted of a 4,4-bis(carbazol-9-yl)biphenyl (CBP) host doped with a 50% fluorescent sky-blue emitter 10,10′-(9-butyl-9*H*-carbazole-3,6-diyl)bis(9-(2-ethylhexyl)-9*H*-pyreno[4,5-d]imidazole) (DK-3) [31], and a 0.1% phosphorescent red emitter Os(fptz)_2_(PPh2Me)_2_ (fptz = 3-trifluoromethyl-5-pyridyl-1,2,4-triazole) [32,33,34].

The fabrication process included firstly spin-coating an aqueous solution of PEDOT:PSS at 4000 rpm for 20 s to form a hole injection layer on a pre-cleaned ITO anode. Before depositing the emissive layer, the solution was prepared by dissolving the host and guest molecules in toluene at 70 °C for 0.5 h with stirring. The resulting solution was then spin-coated at 2500 rpm for 20 s under nitrogen. Following were the depositions of the electron-transporting layer TPBi, the electron injection layer LiF, and the cathode Al by thermal evaporation at 1 × 10^−5^ Torr.

The luminance, spectrum, and Commission Internationale de l’Eclairage chromatic coordinates results, as shown in Table 1, were measured by using a PR655 spectroradiometer, and a Keithley 2400 electrometer was used to measure the current-voltage (I-V) characteristics.

**Table 1:** Effects of doping concentration of the blue and red emitters on the photosynthetic action spectrum resemblance (SR_PAS_), power efficiency (PE), current efficiency (CE), external quantum efficiency (EQE), and the CIE coordinates of the PAS-mimicking OLED devices studied.

Doping Concentration (wt %)		SR_PAS_	PE (lm·W^−1^)	CE (cd·A^−1^)	EQE (%)	1931 CIE Coordinates	Maximum Luminance (cd/m^2^)
Blue emitter	Red emitter		@100 cd/m^2^				
3	0.1	64	0.9	2.0	2.3	(0.43, 0.21)	1109
	0.5	49	1.6	3.4	3.2	(0.59, 0.31)	2031
	1.0	44	1.3	2.8	2.5	(0.64, 0.33)	2454
25	0.1	79	1.3	2.7	2.7	(0.40, 0.22)	1386
50		84	1.7	3.3	3.0	(0.41, 0.25)	1377

## 3. Theory

The SR_PAS_ of a given light source is calculated on the basis of the same energy $\int^{\text{​}}P_{PAS}\left(\text{λ}\right)\text{dλ}$ and it is defined as the following:

(1) $${\text{SR}}_{PAS}=\frac{\int^{\text{​}}P\left(\text{λ}\right)\text{dλ}}{\int^{\text{​}}P_{PAS}\left(\text{λ}\right)\text{dλ}}\;\times 100\%$$

where $P_{PAS}\left(\text{λ}\right)$ is the power distribution of the photosynthetic action spectrum and λ is the wavelength, while

(2) $$P\left(\text{λ}\right)=\{\begin{array}{c} \alpha P_{l}\left(\text{λ}\right)\;if\;P_{PAS}\left(\text{λ}\right)>\alpha P_{l}\left(\text{λ}\right)\\ P_{PAS}\left(\lambda \right)\;if\;P_{PAS}\left(\lambda \right)\leq \alpha P_{l}\left(\text{λ}\right) \end{array}\;$$

where *P_l_* (λ) is the entire power spectrum of the given light source, and α is an arbitrary normalization constant, defined as the following

(3) $$\text{α}=\frac{\int^{\text{​}}P_{PAS}\left(\text{λ}\right)\text{dλ}}{\int^{\text{​}}P_{l}\left(\text{λ}\right)\text{dλ}}\;$$

## 4. Results and Discussion

**Figure 2:**
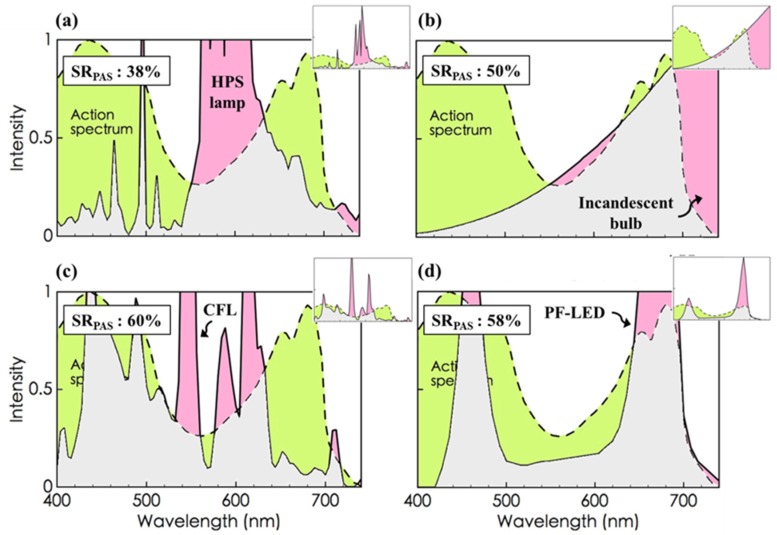
Spectrum resemblance with respect to the photosynthetic action spectrum (PAS), SR_PAS_, for the current lighting devices, including (**a**) a high pressure sodium (HPS) lamp; (**b**) an incandescent bulb; (**c**) a compact fluorescent lamp (CFL); and (**d**) a plant factory light-emitting diode (PF-LED). The SR_PAS_ can also be evidenced by the overlapping area shown in grey, where the area under the dash curve (in green) is for the action spectrum and that under the solid curve (in pink) is for the compared light source. Insets show the entire emissive spectra of the current light sources. The action spectrum data was adopted from *Concepts in Photobiology:* *Photosynthesis and Photomorphogenesis* [9]. The PF-LED data was adopted from the LumiGrow ES330 LED Grow Light Spectrum.

Figure 2 compares the emissive spectra of the current light sources, *i.e.*, (a) high pressure sodium lamp, (b) incandescent bulb, (c) compact fluorescent lamp (CFL), and (d) plant factory light-emitting diode (PF-LED), as also shown in Table S2, with the photosynthetic action spectrum. The calculated PAS resemblance, SR_PAS_, is 38% for the HPS lamp, 50% for the incandescent bulb, 60% for the CFL, and 58% for the PF-LED.

On average, none of the resemblance is high enough to warrant an effective utilization of the given power due to the significantly low spectral match between the light sources and PAS, as indicated by the relatively small overlapping areas shown in grey. In order to prevent the waste of energy, the resemblance should be higher. Moreover, it is surprising to see that the plant growth LED (58% SR_PAS_) does not show any better resemblance than the CFL (60% SR_PAS_), which may indicate that the LED lamp, which is specifically designed for plant growth, is not necessarily more energy-saving than the typical CFL. However, LED still possesses one advantage over the other lighting measures: its spectrum is easily tailored whenever different PASs may be needed for growing different parts of different plants in various seasons [35,36,37,38,39,40,41,42,43,44,45,46,47].

**Figure 3:**
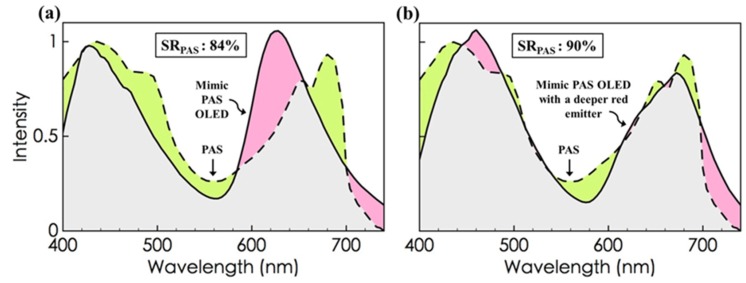
The resulting mimic PAS OLED device shows (**a**) an 84% resemblance with the photosynthetic action spectrum, which increases to (**b**) 90% as a deep red emitter is incorporated further.

Figure 3a shows the spectrum of a mimic PAS OLED with an 84% resemblance with the photosynthetic action spectrum. It is noteworthy that plants do absorb green light to some significant extent, e.g., the absorption of green emissions at 555 nm, for example, is 26% of the peak absorption in the PAS. Furthermore, the energy absorbed in the green light region, *i.e.*, from 495 to 570 nm, measures 17% of the total energy absorbed by the photosynthetic action spectrum. This implies that the green light-dominant mid-wavelength emission is not to be ignored in plant growth [48].

The high spectral resemblance may be attributed to the employment of a twin-peak blue emitter that generates two broad-bands covering the short- to mid-wavelength regions, and the employment of a diffused mono-peak red emitter that generates a relatively wide broad-band extending from the mid- to long-wavelength regions. In addition, an over-90% spectrum resemblance can also be obtainable, provided a deeper red emitter is incorporated, as shown in Figure 3b.

**Figure 4:**
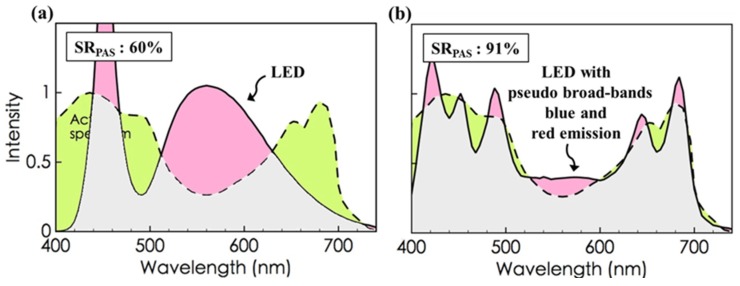
(**a**) A typical LED lamp shows a SR_PAS_ of 60%, which (**b**) can be markedly improved to 91%, as two additional blue and red LEDs peaking at the vicinity of the respective absorption peaks of the PAS are incorporated.

It is interesting to find that the typical LED lamps (Figure 4a) show a SR_PAS_ higher than that of the plant growth-specific LED. That is because the light sources of the former emit a broad band of light ranging from 470 to at least 780 nm and, hence, a much wider overlap with the PAS results in the mid-wavelength region, although the overlap is somewhat lower in the red emission.

To improve on this, the inclusion of more red and blue emissions are suggested in typical white LED lamps. For example, the SR_PAS_ can be increased from 60% to 91% as two additional blue and red LEDs peaking at the vicinity of the respective absorption peaks of the PAS are employed. (Figure 4b).

The resulting OLED light source shows an 84% resemblance with the photosynthetic action spectrum as a twin-peak blue dye, which emits short- to mid-wavelength regions, and a diffused mono-peak red dye, which emits mid- to long-wavelength regions, are employed, and the resemblance can be further improved to over 90% as an additional deeper red emitter is added. For a typical LED, the spectrum resemblance can be improved to 91% as the original single-narrow-band blue emission is replaced by a triple-narrow-band blue counterpart, and an additional double-narrow-band red emission is incorporated. The present approach may facilitate either an ideal use of energy applied for plant growth and/or the design of a better light source for growing different plants. 

## 5. Conclusions

To conclude, we demonstrate a concept for the design of any solid-state lighting technology-based plant growth light sources with an emission closely mimicking the absorption spectrum of plants. The principle is to produce broad-band emissions over the entire absorption spectrum. Taking the photosynthetic action spectrum, for example, the mimicking emission must be high and diffused in the short- and long-wavelength regions, while low but also diffused in the mid-wavelength counterpart. Experimentally, an 84% photosynthetic action spectrum resemblance is obtained by doping a blue fluorescent emitter with diffused twin-emission peaks and a red phosphorescent emitter with a diffused mono-peak, based on an organic light-emitting diode fabrication technology, into a molecular hosting material. This organic LED-based plant growth light source can also show a resemblance of greater than 90% as an additional deeper red emitter is added. However, the resultant maximum luminance was only 1400 cd/m^2^. To carry out a plant growth experiment, a brighter device is required. Potentially, the spectrum resemblance can also be markedly improved to 91%, for example, for a typical LED if two additional blue and red LEDs peaking at the vicinity of the respective absorption peaks of the PAS are employed.

## Supplementary Materials

Supplementary materials can be accessed at: http://www.mdpi.com/1996-1944/8/8/5265/s1.
