# Supplementary material for: Plant Growth Absorption Spectrum Mimicking Light Sources
Source: Materials (Basel). 2015 Aug 13;8(8):5265–75. doi: 10.3390/ma8085240 (PMC5455503; doi:10.3390/ma8085240)
Supplement: Supplementary file 1 [file materials-08-05240-s001.pdf]

Supplementary Materials

Table S1. Summary of the spectrum resemblance with respect to the photosynthetic action spectrum (PAS), SR_{PAS}, for a high pressure sodium (HPS) lamp, an incandescent bulb, a fluorescent lamp, and a plant factory light emitting diode (PF-LED).

**Light Sources**	**SR_{PAS} (%)**
High pressure sodium lamps (HPS)	38
Incandescent bulb	50
Fluorescent tube	60
Plant factory light emitting diode (PF-LED)	58

Table S2. Summary of model number, CCT, and CRI for a high pressure sodium (HPS) lamp, an incandescent bulb, a fluorescent lamp, a plant factory light emitting diode (PF-LED), and a typical LED.

**Light Sources**	**Model No.**	**CCT**	**CRI**
HPS	Street lamp	1760 K	16
Incandescent bulb	(TOA)E27/40W	2300 K	99
CFL	TL5HE35W	5500 K	85
PF-LED	adopted from LumiGrow ES330 LED Grow Light Spectrum (LumiGrow, Novato, CA, USA)		
Typical LED	PH520102	6200 K	77
